# Vision-related Quality of Life after Bilateral Implantation of Monofocal and Multifocal Intraocular Lenses

**DOI:** 10.18502/jovr.v17i1.10166

**Published:** 2022-01-21

**Authors:** Shahram Bamdad, Seyyed Ahmad Razavizadegan, Mohsen Farvardin, Sahar Mohaghegh

**Affiliations:** ^1^Poostchi ophthalmology research center, Department of Ophthalmology, School of Medicine, Shiraz University of Medical Sciences, Shiraz, Iran; ^2^Department of Optometry, School of rehabilitation, Shahid Beheshti University of Medical Sciences, Tehran, Iran

**Keywords:** Cataract Surgery, Multifocal IOL, Quality of Life, Presbyopia, Vision-related Quality of Life

## Abstract

**Purpose:**

To evaluate vision-related quality of life in two sets of patients after routine cataract surgery implanting with traditional versus multifocal intraocular lens (IOLs).

**Methods:**

In a cross-sectional prospective study, 58 and 33 candidates for cataract surgery were divided into traditional (Acrysof SN60WF, Alcon Laboratories, Inc) and multifocal IOL (AcrySof IQ PanOptix IOL TFNT00, Alcon Laboratories, Inc.) groups, respectively. The primary outcome was VFQ-25 scores. The secondary outcomes were making comparisons between the two IOL types in the near vision and the driving items.

**Results:**

mean patients' age in traditional and multifocal IOL groups was 60.85 ± 7.40 (55% female) and 59.85 ± 8.95 (36% female) years, respectively. The mean VFQ-25 total scores in traditional and multifocal IOL groups before and after surgery were 63.69 ± 4.95 and 72.15 ± 9.66, and 98.08 ± 0.70 and 95.70 ± 1.30, respectively (*P =* 0.001 & 0.001). The mean scores of night driving in traditional and multifocal IOL groups were 38.79 ± 20.50 and 44.35 ± 21.12 (*P *= 0.1) before surgery which improved to 97.41 ± 7.68 and 56.45 ± 11.12 after surgery, respectively (*P = *0.001). The mean scores of near vision in traditional and multifocal IOL groups were 46.83 ± 10.56 and 50.53 ± 8.58 (*P *= 0.2) before surgery which improved to 89.94 ± 4.87 and 100.00 ± 0.00 after surgery, respectively (*P *= 0.001).

**Conclusion:**

Vision-related quality of life after cataract surgery with either type of traditional or multifocal (PanOptix) IOLs improved to an excellent level. Traditional IOLs provided more satisfaction in nighttime driving while multifocal IOLs provided increased satisfaction in near and intermediate vision.

##  INTRODUCTION

Cataract is amongst the most prevalent causes of visual impairment in the world.^[1]^ The prevalence of senile cataract increases with age during the years of presbyopia.^[2]^ Cataract surgery with an intraocular lens (IOL) implantation is one of the most common and thought to be one of the most effective surgical procedures in any field of medicine.^[1,2]^ Monofocal lens as the first generation and most common type of intraocular lenses brings far distance into clear focus.^[2]^ However, the ideas of accommodative^[3]^ and multifocal intraocular lenses^[4]^ developed due to increasing demand for activities that require near sight adjustment in the modern life style. Although the progress in the area of accommodative intraocular lenses did not advance as quickly as its counterparts,^[3]^ the multifocal intraocular lenses progressed to a clinically satisfactory level and spectacle independency.^[4,5,6,7,8,9,10,11]^

The trifocal intraocular lenses are the most recent type of multifocal intraocular lenses that provide clear vision not only for far and near distances but also for intermediate distances that facilitate vision for electronic devices and automobile dashboards.^[10]^ Trifocal intraocular lenses that are mostly pupil independent, designed to encompass the refractive–diffractive functionality in addition to extended depth of focus optics^[8]^ which are mostly pupil independent, provide highly successful clinical outcomes,^[5,6,7,8,9,10,11]^ increased visual satisfaction,^[10,11,12]^ and improved quality of life as a result of enhanced vision.^[11,12,13,14,15]^ The trifocal intraocular lens of PanOptix (AcrySof IQ PanOptix intraocular lens TFNT00, Alcon Laboratories, Inc.) is a noble diffractive non-apodized pupil independent aspheric intraocular lens that provides clear vision in 40 cm, 60 cm, 120 cm, 4 m, and optical infinity distances.^[10]^ Some of the previous studies showed that PanOptix provides increased satisfaction for near and intermediate vision patients as compared to other competing tri-focal intraocular lens brands.^[8,10,11]^

Photic phenomena which is defined as light scattering, halos and glare, presents itself when positioned in poor lighting environments. They are known to be the source of minor visual dissatisfaction that may occur after tri-focal intraocular lens implantation.^[14,15,16]^ Photic phenomena occurs as a result of multiple diffraction of the light beam due to the optics of the intraocular lenses. While present in an environment of glare lighting, the optical bench evaluation revealed a decrease in the image quality of the multifocal when compared to the monofocal intraocular lens.^[16]^ Hence, it is of critical importance to evaluate the extent of the effect of these photic phenomena on the patient's vision in order to present a more complete judgement about the patient's state of vision after multifocal intraocular lens implantation.

Since patient-reported outcomes are of paramount importance when evaluating the success of treatments,^[17]^ the first aim of the current study was to evaluate changes in vision as it related to the quality of life after bilateral traditional (monofocal) and multifocal (PanOptix) intraocular lenses implantation in the Iranian population. In this study, we used the Persian-version of the visual function questionnaire-25 (VFQ-25) which had been previously validated in the Persian population.^[18]^ This questionnaire has been widely utilized for evaluating the quality of life in cataractous patients.^[14,19,20]^ Quality of life measurements evaluates the level of improvement that a specific treatment may bring about in various life dimensions beyond the clinical evaluations. The second and third aims of the current study were to compare the near vision and driving items scores cumulated from the questionnaire between the two intraocular lens types.

##  METHODS

This cross-sectional prospective study was conducted at Khalili Eye Hospital, Shiraz, Iran. The study protocol followed the tenets of the Helsinki and was approved by the local ethics committee of Shiraz University of Medical Sciences. Informed consent was obtained from all patients. Inclusion criteria were patients with bilateral cataract and corneal astigmatism < 1.25 diopter power. In addition, patients who routinely drive were included. Exclusion criteria were prior history of refractive surgery, and other ocular conditions that may affect the vision such as glaucoma, age-related macular degeneration, and diabetic retinopathy. Prior to the surgery, optical coherence tomography was performed to exclude patients with any impairment in the macular area such as age-related macular degeneration disease, drusen, and diabetic exudate. Corneal topography was also assessed for all patients to exclude patients with irregular or skewed astigmatism. The excluded patients were not good candidates for multifocal intraocular lens implantation. Uncorrected distance visual acuity, best-corrected distance visual acuity, refraction, slit-lamp bio-microscopy and fundoscopy were performed at baseline and two months after surgery. The postsurgical examinations were performed for all patients in each follow-up session.

Keratometry and axial length measurements with intraocular lens Master 500 (Carl Zeiss Meditec, Germany) was performed prior to the surgery for all patients. The intraocular lens dioptric power was selected to target emmetropia using the intraocular lens power that corresponded to the negative (myopic) predicted refractive outcome closest to zero. The appropriate formulas that corresponded to axial length were used for the intraocular lens power calculation. Patients underwent the intracapsular cataract extraction for the first eye. Two months after the first eye healed, the second eye surgery was performed. All surgical procedures were performed by the same surgeon (FM). According to the patients' visual demand and desire expressed in the consultation session, either the monofocal intraocular lens with the double haptic aspheric design (Acrysof SN60WF, Alcon Laboratories, Inc.) or the multifocal intraocular lens (AcrySof IQ PanOptix intraocular lens TFNT00, Alcon Laboratories, Inc.) was chosen for implantation. It was impossible to randomize patients between these two groups because of the differences in the costs of the intraocular lens and the lifestyle of the patients.

### PanOptix Intraocular Lens

The multifocal intraocular lens of PanOptix is an aspheric, hydrophobic intraocular lens with a blue filter and a 6.0-mm optical zone composed of a 4.5-mm large diffractive area with 15 diffractive zones and an outer refractive rim. It has three focal points from distance to intermediate and near ranges, dividing the incoming light to create intermediate and near add powers of 2.17 diopters (D) and 3.25 D, respectively. Therefore, it provides optimal close reading distances at 60 cm and 42 cm. This novel diffractive structure provides high light utilization transmitting 88% of light at the simulated 3.0 mm pupil size to the retina. This light energy is distributed 25% each for near and intermediate and 50% for distance vision.

### The Persian-Version-VFQ-25

We used the 25-item Persian-version-VFQ, short-form version of 51-item VFQ. It was divided into 12 subscales including general health (two items), general vision (two items), ocular pain (two items), near vision (six items), distance vision (six items), vision-specific social functioning (three items), vision-specific mental health (five items), vision-specific role difficulties (four items), vision-specific dependency (four items), driving (three items), color vision (one item), and peripheral vision (one item). Subscale responses were graded 0 to 100, higher VFQ scores represent a better quality of life. The items were averaged to form subscales, and the sum of averages resulted in the total score. The Persian-version-VFQ-25 questionnaire, as determined in the Iranian population, has been shown to be valid and reliable. Through face-to-face interview by an ophthalmic technician the Persian-version-VFQ-25 results were obtained from all patients at baseline and two months after the cataract surgery of the second eye. For further comparison, we matched the groups of the monofocal and PanOptix intraocular lenses according to the scores of the near vision and driving items at baseline.

### Statistical Analysis

Statistical analysis was performed using SPSS 18.0 for Windows (SPSS Inc., Chicago, Illinois, USA). We used descriptive statistics to illustrate the future of the data. The nonparametric test of Wilcoxon and Mann–Whitney U-test were used to make a comparison between the before and after data within the two groups. *P* < 0.05 was considered as statistically significant.

##  RESULTS

Fifty-eight patients (55% female [32 out of 58]) with the mean age 60.85 ± 7.40 years and 33 patients (36% female [12 out of 33]) with the mean age 59.85 ± 8.95 years were included in the monofocal and PanOptix intraocular lenses groups, respectively. The mean binocular uncorrected distance visual acuity in the monofocal intraocular lens group was 1.24 ± 1.89 and 0.07 ± 0.04 before and after surgery, respectively, (*P = *0.00) and in the PanOptix group was 0.79 ± 1.08 and 0.00 ± 0.00 before and after surgery, respectively (*P = *0.00). The mean scores of the Persian-version-VFQ-25 in the monofocal and PanOptix intraocular lens groups before and after the cataract surgery are presented in Tables 1 and 2.

The mean scores of near vision and driving references in both groups are presented in Graph 1. The driving reference is evaluated in the categories of day and nighttime driving and vision in driving in adverse conditions. Two groups were matched in the mean scores of near vision driving references before surgery (*P* > 0.05). The differences in the references of near vision, nighttime driving, and vision in driving in adverse conditions were statistically significant between the two groups (*P = *0.001).

There were no intra- or postoperative complications or adverse effects in the study population in the two groups.

**Table 1:** Vison-related quality of life in patients with monofocal intraocular lens


	**Mean ± Std. Deviation ** ***N*** ** = 58**	**Mean ± Std. Deviation ** ***N*** ** = 58**	***P*** **-value**
General health	47.84 ± 17.69	100.00 ± 0.00	0.001
General vision	54.31 ± 14.91	88.91 ± 12.47	0.001
Peripheral vision	50.43 ± 12.81	100.00 ± 0.00	0.001
Color vision	65.08 ± 12.33	100.00 ± 0.00	0.001
Mental health	77.58 ± 7.68	100.00 ± 0.00	0.001
Ocular pain	71.87 ± 15.23	88.36 ± 8.06	.001
Near vision	46.83 ± 10.56	89.94 ± 4.87	0.001
Distance vision	55.02 ± 7.47	100.00 ± 0.00	0.001
Social function	70.47 ± 13.18	100.00 ± 0.00	0.001
Role limitation	86.63 ± 8.71	100.00 ± 0.00	0.001
Dependency	91.66 ± 6.24	100.00 ± 0.00	0.001
Driving	47.27 ± 15.48	98.27 ± 4.05	0.001
Total Score	63.69 ± 4.95	98.08 ± 0.70	0.001

**Table 2:** Vison-related quality of life in patients with PanOptix intraocular lens


	**Mean ± Std. Deviation (before) ** ***N*** ** = 33**	**Mean ± Std. Deviation (after) ** ***N*** ** = 33**	***P*** **-value**
General health	74.24 ± 14.63	96.96 ± 8.28	0.001
General vision	69.69 ± 14.99	93.18 ± 11.30	0.001
Peripheral vision	64.39 ± 12.54	94.69 ± 10.37	0.001
Color vision	100.00 ± 0.00	100.00 ± 0.00	> 0.99
Mental health	69.69 ± 14.58	89.77 ± 9.08	0.001
Ocular pain	94.50 ± 6.00	100.00 ± 0.00	0.01
Near vision	51.01 ± 9.02	100.00 ± 0.00	0.001
Distance vision	77.02 ± 12.50	100.00 ± 0.00	0.001
Social function	88.25 ± 10.33	96.59 ± 3.16	0.001
Role limitation	68.56 ± 20.99	100.00 ± 0.00	0.001
Dependency	69.69 ± 17.77	100.00 ± 0.00	0.001
Driving	44.08 ± 21.10	77.15 ± 3.70	0.001
Total	72.15 ± 9.66	95.70 ± 1.30	0.001

**Figure 1:**
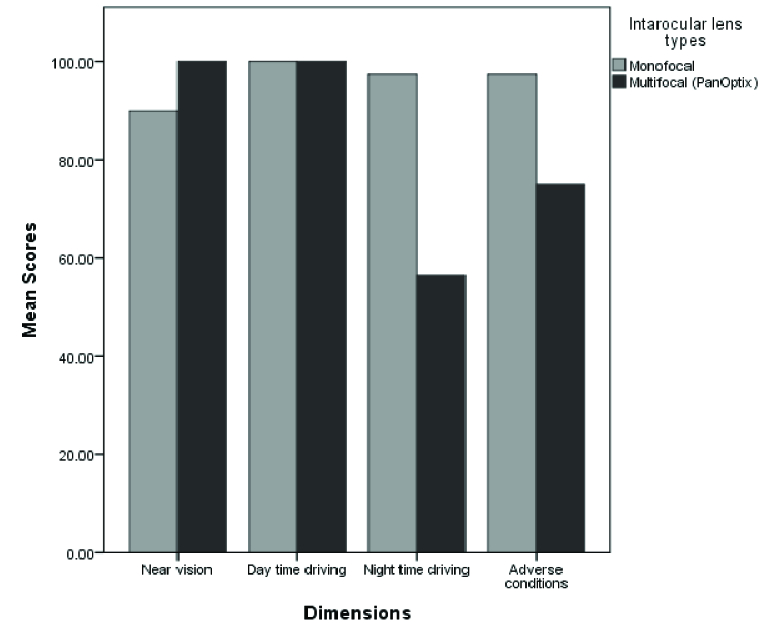
A comparison between monofocal and PanOptix intraocular lenses.

##  DISCUSSION

Nowadays, cataract surgery has the potential to provide excellent visual rehabilitation for all of the far, intermediate, and near distances by implanting multifocal intraocular lenses. However, monofocal intraocular lenses are still the most commonly used types of intraocular lenses across the world. Multifocal intraocular lenses have the potential to provide spectacle independency for pan-distances while they may increase the possibility of minor photic phenomena occurring due to their optical designs. Previous clinical evaluations revealed that a high level of visual acuity and contrast sensitivity after multifocal intraocular lens implantation is obtainable. However, the mesopic contrast sensitivity in patients with multifocal intraocular lenses has been reported to be less than monofocal.^[16]^ In the current study using the Persian-version-VFQ-25, we evaluated the state of improvement that cataract surgery by implanting monofocal and multifocal (PanOptix) intraocular lenses may bring about in the patients' quality of life.

Our data analysis shows that the vision-related quality of life improved to an excellent level after cataract surgery with either type of monofocal or PanOptix intraocular lenses. For both groups, the mean score values in all dimensions were approximately > 90, except for the driving reference in the PanOptix group (=75) that is discussed thoroughly in the paragraphs below. The prominent improvements observed in the dimensions of general and mental health, social function, dependency, and role limitation reveal that cataract surgery with either type of intraocular lenses improves both vision and sense of well-being. Cataract surgery with either lenses reduces the patients' stress level, and improves the patients' social relationships and activities. Our results are in agreement with the results of previous studies.^[11,13,14,21]^ Akman et al^[13]^utilized the VF-14 quality-of-life questionnaire and evaluated the vision-related quality of life in 48 patients after PanOptix implantation. Similarly, they reported an overall high level of satisfaction and a high quality of life among these patients. In another study, Carneros-Llorente et al^[11]^ compared the visual acuity, contrast sensitivity, and quality of life scores among three trifocal designs. They reported high satisfaction levels with all of the designs and a slightly improved intermediate vision with PanOptix. Hamidi et al^[21]^ reported highly satisfactory results with the toric PanOptix intraocular lens model in the eyes with longer or shorter axial length.

The novelty of the PanOptix intraocular lens is the provision of clear images at near and intermediate distances. Since the PanOptix group obtained the maximum score in the “near & intermediate fine works” category, it is evident that the PanOptix design from the patients' viewpoint was very successful in achieving its aim. Although patients with a monofocal intraocular lens may take advantage of near glasses and obtain a good score the spectacle independency that PanOptix provides has resulted in a prominently higher score in these patients. Our results are in agreement with the results of previous studies.^[22,23,24]^ Chichester et al reported spectacle independence in 90% of patients with trifocal intraocular lenses, and all patients stated that they would be willing to repeat surgery with the same intraocular lens type.^[22]^ Akman et al^[13]^reported no difficulty in near activities such as reading books and phone numbers, writing checks, performing computer tasks, wearing make-up, and cooking with PanOptix. However, they reported a little difficulty in reading very small print sizes and sewing. Rementería-Capelo et al^[15]^ reported high scores in near activities such as reading newspapers, viewing prices on goods, and doing handcrafts.

Our findings revealed that nighttime driving and driving in adverse conditions had a moderate improvement in the PanOptix group. Albeit the daytime driving reached the maximum level of improvement in this group. We relate that to the diffractive design of the PanOptix lens and the light halos that appears in the field.^[16,25]^ Trifocal PanOptix intraocular lens provides three images simultaneously, so in poor lighting conditions, the multiple images' shadows disturb the vision. It seems that the effect of shadows is significant in nighttime driving and is reflected in the patient's reported outcome. Moreover, the higher-order aberrations amount increases at nights due to the larger pupil size, that superimposes the previous problems of halos and glare with this design. Although the results of our study were in agreement with previous studies,^[12,13]^ it has been reported that as a result of neural adaptation this problem described above decreases with time.^[26]^ Previous studies revealed that resolving the remaining uncorrected refractive errors with wavefront-guided lasers in situ keratomileuses can prominently enhance the satisfaction level of patients after multifocal lens implantation.^[27]^ Moreover, performing the incision after the corneal steep axis in surgery results in astigmatism reduction in the corneal, < 1D power. Consequently, this consideration with PanOptix design improves the quality of vision and the satisfaction of the patients.^[28]^

As expected, postoperative uncorrected visual acuity prominently improved after cataract surgery with both lens types and no adverse effects were observed in both groups. Previous studies revealed that presurgical visual acuity should not be considered as the only indicator when performing cataract surgery. Rather, vision-related quality of life measurements with validated questionnaires is recommended as a necessary additional tool for making decisions about surgery.^[29]^ These tools are necessary because there might be high visual demands present in some patients' lifestyles where even early stage cataract can profoundly degrade their quality of life and vision satisfaction. In choosing the intraocular lens design patients who are more demanding and are perfectionists may request multifocal lenses while the more easygoing patients may choose the traditional design. Therefore, the personality of the patient itself may affect postoperative acceptance. In our study, we observed increased satisfaction from patients in near vision with PanOptix. However, if the traditional group were served by PanOptix, this resultant satisfaction may not be obtained, as PanOptix intraocular lens requires making an adjustment for the near vison which easygoing patients may find inconvenient.

The strength of our study was utilizing the Persian-version VFQ-25 as a validated questionnaire which is widely used in cataract evaluations^[14,19,20]^ in global populations.^[30,31,32]^ Thus, it makes comparisons in future studies more convenient than using self-designed questionnaires. The study's limitation was the short follow-up time. A longer follow-up period is recommended when considering the probability of neural adaptation in evaluating the state of nighttime driving after PanOptix intraocular lens implantation. Another limitation was not measuring and comparing the contrast sensitivity between the two groups. In addition, our study does not cover the high myopic and extremely hyperopic eyes. Previous studies show satisfactory clinical outcomes in such cases, however less successful results when compared to patients with more normal refractive errors.^[33,34]^ Therefore, in order to properly address these limitations that exist, more resources are required in determining the patients' vision satisfaction and reported outcomes in such cases.

In conclusion, we discovered that in all aspects of life both monofocal and PanOptix intraocular lens provide patients with a substantial level of vision-related quality of life and visual satisfaction. The monofocal intraocular lenses enhanced the vision of the patient during nighttime driving as compared to that of the PanOptix multifocal intraocular lens. In addition, the PanOptix intraocular lens improved the quality of the vision of the patients in the near and intermediate categories as a result of its optical design. Therefore, it is recommended in order to adequately assess choosing the suitable option it is mandatory to consider the patient's visual demand prior to the cataract surgery and inform the patient about the probability of visual dissatisfaction occurring with nighttime driving if multifocal intraocular lenses are the chosen option.

##  Financial Support and Sponsorship

Nil.

##  Conflicts of Interest

None declared.
